# Burden of substance use and correlates among engineering students

**DOI:** 10.1192/j.eurpsy.2026.10893

**Published:** 2026-07-31

**Authors:** S. Bradai, N. Ketata, H. Ben ayed, M. Ben Hamida, M. Kassis, J. Jedidi, S. Yaich

**Affiliations:** 1 Hedi Chaker hospital; 2 Habib bourguiba hospital; 3 faculty of medicine of sfax, sfax, Tunisia

## Abstract

**Introduction:**

The use of tobacco and other substances remains a major public health concern affecting all age groups and generations. Young adulthood represents a peak period for the emergence of such behavioral problems, particularly among university students, including those in engineering.

**Objectives:**

This study aimed to explore the prevalence of substance use among engineering students (ES) in Sfax, Southern Tunisia and its associated factors.

**Methods:**

It was a cross-sectional study conducted during the 2024-2025 academic year among ES of all levels. It was conducted in Sfax, South of Tunisia by using the stratified random sampling method. Data collection was performed using an anonymous self-administered questionnaire.

**Results:**

Overall, 302 ES were enrolled in the study. The median age was 23 years with an interquartile range (IQR)= [21-24 years]. There were 148 females (60.9%), giving a male to female ratio of 0.64. History of chronic disease was noted in 41 ES (13.6%). There were 33 ES (10.9%) having low monthly income and 53 ES (17.5%) lived in rural areas. Academic failure during the last year was noted among 38 ES (12.6 %). As for lifestyle behaviours, a total of 61 ES (14.9%) practised physical activity and 261 ES (86.4%) had leisure activities.

Prevalence of tobacco use, alcohol consumption and illicit drug use among ES were 23.5% (n=71), 16.2% (n=49) and 4.6% (n=14), respectively.

Prevalence of tobacco use was statistically associated with male gender (OR=3.407; p=<0.001), living in rural areas (OR =1.280; p=<0.001) and academic failure (OR=1.256; p=<0.001). In addition, it was statistically associated with alcohol consumption (OR =2.496; p=<0.001) and illicit drug use (OR =1.219; p=<0.001). Besides, having a leisure activity was statistically associated with lower prevalence of tobacco use among ES (OR=0.87; p=0.029).

**Conclusions:**

A noteworthy finding was the high prevalence of both tobacco use and co-addictions among engineering students, with lifestyle behaviors and socioeconomic status identified as key predictors. These results emphasize the importance of implementing monitoring and support programs to address existing addictions and prevent the initiation of new substance use.

**Disclosure of Interest:**

None Declared

